# Lumasiran treatment in pediatric patients with PH1: real-world data within a compassionate use program in Italy

**DOI:** 10.1093/ckj/sfae090

**Published:** 2024-03-28

**Authors:** Francesca Taroni, Licia Peruzzi, Germana Longo, Francesca Becherucci, Gabriele Malgieri, Maria Michela D'Alessandro, Giovanni Montini

**Affiliations:** Pediatric Nephrology Dialysis and Transplant Unit, Fondazione IRCCS Ca’ Granda Ospedale Maggiore Policlinico, Milan, Italy; Pediatric Nephrology Dialysis and Transplant Unit, Regina Margherita Children's Hospital, Torino, Italy; Pediatric Nephrology, Dialysis and Transplant Unit, Department of Woman and Child Health, Azienda Ospedaliera-University of Padova, Padova, Italy; Nephrology and Dialysis Unit, Meyer Children's Hospital IRCCS, Florence, Italy; Paediatric Nephrology, Dialysis and Renal Transplantation Santobono Pausilipon Children's Hospital, Naples, Italy; Pediatric Nephrology Unit, Azienda di Rilievo Nazionale ed Alta Specializzazione (ARNAS) Civico, Di Cristina, Benfratelli, Palermo, Italy; Pediatric Nephrology Dialysis and Transplant Unit, Fondazione IRCCS Ca’ Granda Ospedale Maggiore Policlinico, Milan, Italy; Department of Clinical Sciences and Community Health, University of Milano, Milan, Italy

**Keywords:** lumasiran, outcome, pediatric patients, primary hyperoxaluria type 1

## Abstract

**Background:**

Primary hyperoxaluria (PH) is a rare, severe genetic disorder, characterized by increased urinary excretion of calcium oxalate, which is responsible for kidney damage and systemic clinical manifestations. Since the year 2020, a new molecule, lumasiran, based on RNA interference (RNAi) technology, has been added to the traditional therapeutic approach. The aim of this analysis was to define the baseline characteristics of a PH1 pediatric population treated with lumasiran in a compassionate-use program setting, and to evaluate the medium-term efficacy of this drug in the routine clinical setting.

**Methods:**

A retrospective observational analysis was conducted in nine pediatric patients (male:female 5:4; median age at lumasiran start 1.9 years, range 0–14.1). Data concerning oxalate concentration in plasma and urine, kidney stones events, ultrasound and kidney function were collected during the study period (follow-up, mean ± standard deviation: 15.3 ± 5 months).

**Results:**

In this analysis, a reduction in the urinary oxalate to creatinine ratio (reduction range within the sixth month of treatment from 25.8% to 69.6%, median 51.2%) as well as plasma oxalate concentration under the limit of supersaturation of oxalate in all the patients. Only one patient presented new stone events; kidney ultrasonographic findings related to nephrocalcinosis remained stable in eight out of nine patients. Glomerular filtration rate remained stable during treatment. No adverse events related to lumasiran were noted.

**Conclusion:**

Data from this analysis support the efficacy and safety of lumasiran in a pediatric clinical setting, especially if administrated in early life.

## Graphical Abstract

**Graphical Abstract:**
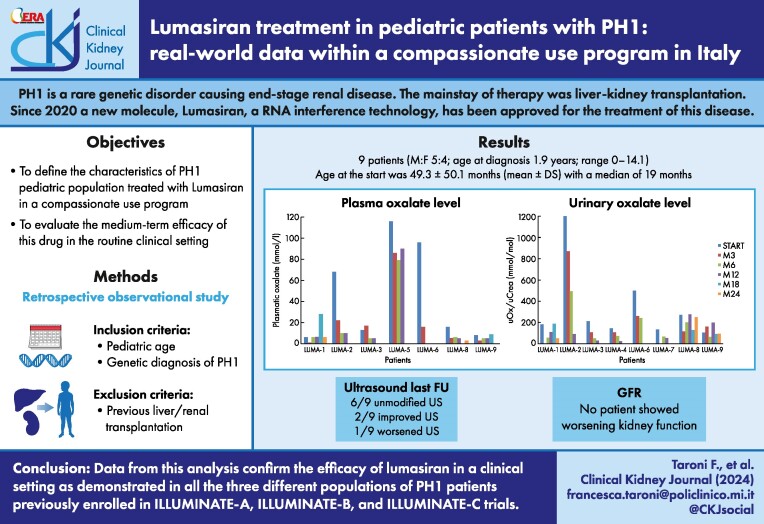

KEY LEARNING POINTS **What was known:**

- Primary hyperoxaluria type 1 (PH1) is a rare genetic disease characterized by recurrent nephrolithiasis, nephrocalcinosis and systemic oxalosis causing end-stage renal disease.
- The mainstay of therapy has been liver–kidney transplantation. Since 2020 a new molecule, lumasiran, an RNA interference technology, has been approved for the treatment of this disease.

**This study adds:**

- ILLUMINATE-A, ILLUMINATE-B and ILLUMINATE-C trials showed the efficacy of lumasiran in the reduction of urinary and plasmatic reduction in patients of different ages and with various stages of chronic kidney disease.
- More data are necessary to evaluate efficacy of this drug in infancy and childhood.

**Potential impact:**

- This paper focuses on pediatric patients affected by PH1, who are a population at high risk of end-stage renal disease.
- The experience about the use of lumasiran in a clinical setting in children could help clinicians to better understand the impact of this drug in childhood.

## INTRODUCTION

Primary hyperoxaluria type 1 (PH1) is a rare autosomal recessive disease characterized by increased renal excretion of oxalate, recurrent nephrolithiasis, nephrocalcinosis and accumulation of insoluble oxalate in the body (systemic oxalosis) [1].

PH1, by far the most common form (80% of cases), is caused by mutations in the *AGXT* gene, which codes for the hepatic enzyme AGT [2].

The characteristic biochemical marker is urinary oxalate hyperexcretion >45 mg (0.5 mmol/L/1.73 m^2^) on 24-h urine collection, or a urinary oxalate to creatinine ratio above the range for age [3].

The disease can begin at any age, from birth to the sixth decade of life, with a median of 5.5 years. The clinical presentation varies from childhood nephrocalcinosis to growth retardation because of chronic kidney damage, to a picture of recurrent or occasional kidney stones in the adult. However, 20%–50% of patients already have advanced or even end-stage kidney damage at the time of diagnosis [4]; moreover, about 10% of them are diagnosed only after disease recurrence in a transplanted kidney [5]. The patient may show early systemic involvement (oxalosis), caused by deposition of insoluble oxalate crystals in the body, resulting in extensive and severe organ damage.

Until 2020, the mainstays of therapy for PH1 were basically three: conservative therapy, dialysis and liver–kidney transplantation. Since 2020, an innovative pharmacological approach, namely lumasiran, has also been added to the therapeutic armamentarium. It belongs to a group of molecules referred to as small interfering RNAs (siRNAs), that is a double-stranded RNA with an interfering role that can modulate hepatic oxalate metabolism [6]. The ILLUMINATE-A, -B and -C studies have provided interesting evidence regarding the efficacy and safety of lumasiran [7–11].

Following Food and Drug Administration and European Medicines Agency approval in November 2020, lumasiran is currently the only major therapeutic approach that has been shown to favorably impact the natural history of PH1.

Here, we present real-world data on the use of lumasiran in a pediatric population treated in various centers in Italy, to evaluate the medium-term efficacy of this drug in the routine clinical setting.

## MATERIALS AND METHODS

### Objectives

The objectives of the present study are: (i) to confirm the reduction of urinary oxalate excretion and plasma oxalate concentration in patients enrolled and treated with lumasiran; (ii) to evaluate in the same patients the ultrasonographic changes in the number of renal stones at specified time points from treatment initiation; and (iii) to identify the number of renal stone events (colic or macrohematuria) and the course of renal function over time.

### Methods

This was a retrospective observational multicentric study conducted on patients who met the following criteria: (i) age 0–18 years at start of treatment; (ii) clinical diagnosis of primary hyperoxaluria type 1 (PH1) with genetic confirmation; and (iii) initiation of treatment with lumasiran within the compassionate-use program setting (CUP). PH1 patients undergoing previous liver/renal transplantation were excluded.

Data were collected anonymously in a digital database and included:

(i) Demographic data (date of birth, gender, geographical origin).
(ii) Diagnosis (event leading to diagnosis, date and age at first visit, genetic analysis).
(iii) Renal function, plasma and urinary oxalate, ultrasonographic data (location and number of stones) at the time of treatment initiation and during follow-up.
(iv) Record of clinical events related to renal stones in the 24 months prior to the initiation of lumasiran and during the treatment.
(v) Treatment (medical, surgical) in the 24 months prior to the initiation of lumasiran.

The assessment of efficacy consisted in the evaluation of urinary oxalate excretion, plasma levels of oxalate, kidney function and the recording of kidney stone events (colic and/or gross hematuria) and kidney ultrasonographic data in routine clinical practice.

Safety was assessed by collecting adverse events (including serious adverse events).

Patients were evaluated at start of lumasiran and at 3, 6, 12, 18 and 24 months after the start of the treatment, respectively, indicated as M3, M6, M12, M18 and M24.

Lumasiran was administered at recommended dose, according to the manufacturer's instructions. The study has been approved by the ethics committee of all participating centers.

## RESULTS

As of June 2023, nine pediatric patients (male:female 5:4; age at diagnosis 1.9 years, range 0–14.1) affected with PH1 were currently in treatment with lumasiran in the participating centers.

Initiation of lumasiran treatment occurred for the first patient enrolled in December 2020, while the last patient started the treatment in January 2022. Lumasiran administration was initiated at an age <2 years in five patients, and at an age between 2 and 18 years in four patients. Patients’ age at the start was 49.3 ± 50.1 months (mean ± standard deviation) with a median of 19 months (youngest patient's age: 11 days; oldest patient's age: 13 years).

All patients received conservative therapy before and during lumasiran administration (hydration, pyridoxine, potassium citrate, sodium bicarbonate and other therapies based on the degree of kidney damage). Only one patient (LUMA-5), given the severity of kidney impairment, was placed on peritoneal dialysis almost simultaneously with the diagnosis; he presented growth impairment but not signs of systemic oxalosis. Growth improved after the start of the peritoneal dialysis from 3 to 10 percentile. Peritoneal dialysis was preferred to hemodialysis in consideration of the age of the patient (0.5 years), to preserve vascular accesses and for logistic reasons because he lived very far from the pediatric dialysis center. None received kidney or liver transplantation during the period of the study.

The demographic and clinical characteristics of the children included in the study are summarized in Table 1.

**Table 1:** Demographic and clinical characteristics of the children included in the study.

Patient	Sex	Birth weight (kg)	Genetic testing	Symptoms at onset	Age at onset (years)	Age at diagnosis (years)	Age at start of lumasiran (years)	Ultrasound at start	Surgery^a^	Kidney stone events^b^
LUMA-1	F	3	c.121G > A (pGly41Arg)	Colic	3.4	3.6	6.4	Bilateral kidney stones	2	2
			c.121G > A (pGly41Arg)							
LUMA-2	F	2.6	c.466G > A (pGly156ARg)	Affected brother	0.3	0	0.0	Normal	0	0
			c.943–1G > T							
LUMA-3	M	2	c.508G > A (p. Gly 170 Arg)	Poor growth	0.4	1.0	1.2	Bilateral kidney stones	0	0
			c.508G > A (p. Gly 170 Arg)							
LUMA-4	F	2.9	c.603C > A (p. Asp201Glu)	Incidental ultrasound	13.1	13.5	14.1	Unilateral kidney stones	0	3
			c.603C > A (p. Asp201Glu)							
LUMA-5	M	3.7	c.33dup (p.Lys12GlnfsTer156)	Incidental ultrasound	0.3	0.5	1.7	Nephrocalcinosis	0	0
			c.508G > A (p. Gly170Arg)							
LUMA-6	M	4.1	c.466G > A (p. Gly156Arg)	Incidental ultrasound	0.1	0.2	0.9	Nephrocalcinosis	0	0
			c.466G > A (p. Gly156Arg)							
LUMA-7	F	3.4	c.(33delC)	Incidental ultrasound	1.0	7.6	9.6	Nephrocalcinosis	0	5
			c.(33delC)							
LUMA-8	M	3	c.33dupC (p. Lys12GInfs156)	Poor growth	0.7	0.8	1.9	Bilateral kidney stones, nephrocalcinosis	0	1
			c.508G > A (p. Gly170Arg)							
LUMA-9	M	3	c.33dupC (p. Lys12GInfs156)	Affected brother	2.7	2.8	4.2	Bilateral kidney stones	0	0
			c.508G > A (p. Gly170Arg)							

^a^Number of surgical procedures for kidney stones in the 24 months prior to the start of treatment with lumasiran.

^b^Number of renal stone events in the 24 months prior to the start of treatment with lumasiran.

F: female; M: male.

### Urinary oxalate values

**Figure 1:**
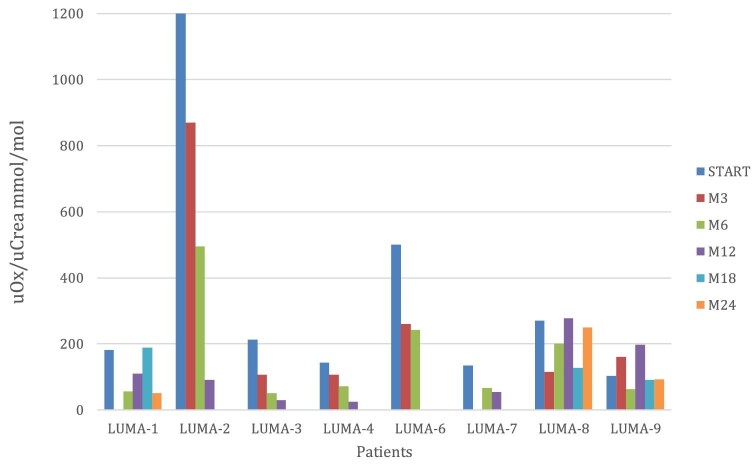
Urinary oxalate value during the follow-up.

As can be seen in Table 2, urinary oxalate levels, expressed as spot urinary oxalate/creatinine ratio (Ox/creat in mmol/mol), were significantly reduced in all patients (>30% from baseline between M3 and M6) after the start of lumasiran (Fig. 1). Patient LUMA-5 was anuric so he could not be tested for urinary oxalate excretion. Variable values of oxaluria were observed in three patients between M12 and M18.

**Table 2:** Urinary, plasmatic oxalate and GFR during the follow-up.

		Urinary oxalate/creatinine mmol/mol (Δ%)^a^						Plasmatic oxalate (mmol/L)						GFR (mL/min/1.73 m^2^)	
Patient	FU	Start	M3	M6	M12	M18	M24	Start	M3	M6	M12	M18	M24	Start	Last FU
LUMA-1	24	182	93 (–48.8)	55 (–69.6)	110 (–39.5)	189 (+4)	50 (–72.5)	6	0.9	6	6.5	28	6	80	80
LUMA-2	12	1200	870 (–27.5)	495 (–58.8)	90 (–92.5)			68	22	10	10			>90	>90
LUMA-3	12	214	107 (–49.9)	50 (–76.6)	30 (–86)			13	17	5	5			>90	>90
LUMA-4	12	143	106 (–25.9)	72 (–49.5)	24 (–83.2)									27	30
LUMA-5	12							116	86	79	90			<15	<15
LUMA-6	12	500	260 (–48)	243 (–51.4)	245 (–51)			96	16					80	80
LUMA-7	12	135		66 (–51.1)	54 (–60)						2			70	70
LUMA-8	24	271	115 (–57.6)	201 (–25.8)	277 (+2.1)	127 (– 53.1)	250 (–7.7)	16	5.4	6	5		3	86	90
LUMA-9	18	103	161 (+56.3)	63 (–38.8)	197 (+91)	90 (–12.6)	92 (–10.7)	8	3	5	5	9		>90	>90

^a^Δ%: percentage change from the initial value.

FU: follow-up (months).

### Plasmatic oxalate values

**Figure 2:**
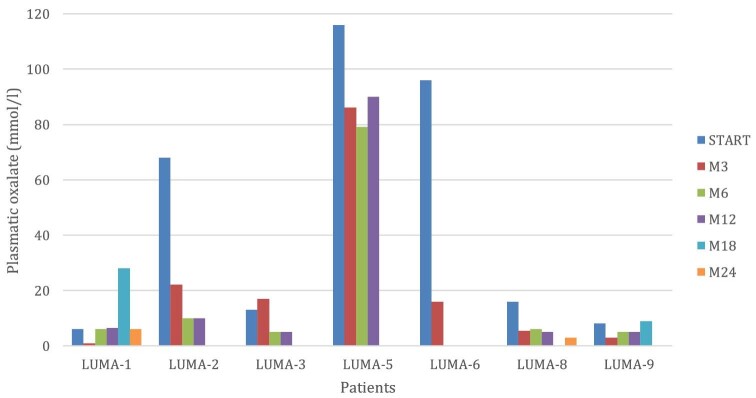
Plasmatic oxalate value during the follow-up.

As described in Table 2, plasmatic oxalate value was over the limit of oxalate supersaturation in three patients (LUMA-2, LUMA-5, LUMA-6) before treatment with lumasiran; at M6 plasma oxalate levels were below the limit of oxalate supersaturation (20 mmol/L) in all treated patients. It should be noted that the only patient who did not reach this target was LUMA-5, who was under renal replacement therapy with peritoneal dialysis for end-stage renal failure. In two patients (LUMA-4 and LUMA-7) oxalemia was not tested during the period of the study (Fig. 2).

### Ultrasound

The ultrasound scan at the start of treatment with lumasiran showed evidence of nephrolithiasis in six patients and nephrocalcinosis in three, while it was completely normal in LUMA-2, who received a prenatal diagnosis for affected brother.

At last follow-up, six patients had an ultrasound scan unmodified, and two patients showed the disappearance of the nephrolithiasis. One patient (LUMA-2) developed nephrocalcinosis at 3 months of age complicated by unilateral nephrolithiasis at 12 months of age.

### Kidney stone events and surgery

Four patients presented with kidney stone events in the 24 months prior to the initiation of lumasiran therapy, with a frequency of one to five episodes (Table 1). No new clinical events attributable to nephrolithiasis were observed in the cohort under analysis during the observation period.

One patient (LUMA-1) received two surgical procedures for kidney stones in the 24 months prior the start of treatment (Table 1). None of the patients needed surgical interventions after starting lumasiran.

### Time course of glomerular filtration rate

Patients enrolled in the study had different stages of chronic kidney disease (CKD) at the start of treatment.

Glomerular filtration rate (GFR) values remained stable during treatment, and no patient showed worsening kidney function (Table 2).

### Tolerability

None of the enrolled patients reported any major adverse events, either clinical or laboratory. Monitoring of liver function showed stability and normality of the biochemical parameters being monitored. The most frequently reported adverse event was erythema at the injection site.

## DISCUSSION

This retrospective study conducted in a cohort of pediatric patients with PH1 receiving lumasiran showed a reduction in the urinary oxalate to creatinine ratio (>30% from the basal value) as well as in plasma oxalate concentration, which remained below the level of supersaturation of oxalate. None of the patients needed surgery for kidney stones during the follow-up and ultrasound scan was unmodified in eight out of nine patients. GFR remained stable during treatment.

The results of this study are consistent with the results of the previous ILLUMINATE-A, ILLUMINATE-B and ILLUMINATE-C trials [7–11] with respect to plasma oxalate levels, urinary oxalate levels and new stone-related events. In fact, the aforementioned studies demonstrated a reduction in plasma and urinary oxalate levels during the months of treatment in patients >6 years of age (ILLUMINATE-A), <6 years of age (ILLUMINATE-B) and patients of all ages with severe renal failure (ILLUMINATE-C), as well as stability of GFR over the study period. Notably, all treated patients showed a rapid decrease in urinary oxalate levels during the first 6 months of treatment, with stabilization of urinary oxalate concentrations in the following months. Similarly, in our cohort plasma oxalate levels decreased rapidly during the months of treatment and remained below the supersaturation threshold for calcium oxalate thereafter. Some patients showed a transient increase of oxaluria after the sixth month of treatment. This data was described in ILLUMINATE trials and attributed to the release of oxalates stored in bone tissue [7–11].

In our cohort, plasma oxalate was increased also in some patients affected by CKD1 (LUMA-2, -3, -8 and -9).

LUMA-2, who presented at birth a very high plasmatic level of oxalate (68 mmol/L), received an antenatal diagnosis of PH1 for affected brother and started lumasiran at 11 days old. So it could be possible to speculate that the high levels of plasmatic oxalate did not cause kidney damage thanks to a tempestive treatment. LUMA-3 -8 and -9 showed a slight increase in plasmatic oxalate (13, 16 and 8 mmol/L, respectively). Elevated oxalate levels in patients with normal GFR have also been described by other authors. The relationship between plasma oxalate and estimated GFR in patients with preserved kidney function is not well established. Hillebrand *et al*. showed that plasma oxalate has a limited validity in PH1 patients with stable kidney function [12]. Another study conducted by Milliner *et al*. on 106 patients affected by hyperoxaluria with GFR >40 mL/min/1.73 m^2^ found a moderate and statistically significant inverse correlation between GFR and plasma oxalate in patients with PH already at early stages of CKD, concluding that a correlation could be present before substantial loss in kidney function occurs [13].

In the patients described in this study, as well as in previous clinical trials, we found a reduction in stone-related events compared with the 2 years prior to treatment. However, kidney ultrasound findings worsened in only one case (LUMA-2). This patient, who was the youngest in the cohort, had received a prenatal diagnosis of PH1 due to family history; although she had completely normal kidney ultrasound at baseline, she showed the onset of nephrocalcinosis at 3 months of age, later complicated by nephrolithiasis, with findings remaining stable in the following months of follow-up. This trend is like that reported by Méaux *et al*. who, in a patient prenatally diagnosed with PH1 and treated with lumasiran from the 9th day of life, showed the presence of nephrocalcinosis on kidney ultrasonography performed after 2 months of follow-up, which subsequently regressed from the 10th month of life [14].

One of the strengths of the present study is that it reports data from “real-world” practice, including patients with heterogeneous characteristics from all the different groups included in the ILLUMINATE trials. All patient types enrolled in the ILLUMINATE trials were included in the present study and the obtained results indicate that lumasiran is effective in clinical practice, including patients with heterogeneous characteristics.

Limitations of the present study include: (i) the small number of patients enrolled, which is too small a sample to determine statistical significance; and (ii) the short duration of follow-up, which precludes the possibility of collecting long-term data on GFR trends.

## CONCLUSIONS

Lumasiran is today the only therapeutic option with real impact on the management of PH1. This retrospective observational analysis provides valuable real-world evidence on the use of lumasiran for the treatment of PH1. Lumasiran reduces plasma and urinary oxalate levels in treated patients and also seems to be associated with stabilization of GFR and improvement of renal stone disease. Furthermore, lumasiran has a good tolerability profile and is not associated with significant adverse events.

## Data Availability

Data available on request.

## ADDENDUM

The authors report that the clinical history of the LUMA-2 patient has also been reported in another publication as a case report [15].
